# Patient influence on general practice service improvement decision making: a participatory research mixed-methods intervention study

**DOI:** 10.3399/BJGP.2023.0263

**Published:** 2024-04-03

**Authors:** Jessica Drinkwater, Anne MacFarlane, Maureen Twiddy, David Meads, Ruth H Chadwick, Ailsa Donnelly, Phil Gleeson, Nick Hayward, Michael Kelly, Robina Mir, Graham Prestwich, Martin Rathfelder, Robbie Foy

**Affiliations:** Centre for Primary Care and Health Services Research, University of Manchester, Manchester, and honorary research fellow, Leeds Institute of Health Sciences, University of Leeds, Leeds, UK.; Participatory Health Research Unit, School of Medicine and Health Research Institute, University of Limerick, Limerick, Ireland.; Hull York Medical School, Institute of Clinical and Applied Health Research, University of Hull, UK.; Leeds Institute of Health Sciences, University of Leeds, Leeds, UK.; Leeds Institute of Health Sciences, University of Leeds, Leeds, UK.; Leeds Institute of Health Sciences, University of Leeds, Leeds, UK.; Leeds Institute of Health Sciences, University of Leeds, Leeds, UK.; Leeds Institute of Health Sciences, University of Leeds, Leeds, UK.; Leeds Institute of Health Sciences, University of Leeds, Leeds, UK.; Leeds Institute of Health Sciences, University of Leeds, Leeds, UK.; Leeds Institute of Health Sciences, University of Leeds, Leeds, UK.; Leeds Institute of Health Sciences, University of Leeds, Leeds, UK.; Leeds Institute of Health Sciences, University of Leeds, Leeds, UK.

**Keywords:** general practice, patient participation, participatory research, primary care

## Abstract

**Background:**

Health policy promotes patient participation in decision making about service organisation. In English general practice this happens through contractually required patient participation groups (PPGs). However, there are problems with the enactment of PPGs that have not been systematically addressed.

**Aim:**

To observe how a co-designed theory-informed intervention can increase representational legitimacy and facilitate power sharing to support PPGs to influence decision making about general practice service improvement.

**Design and setting:**

Participatory action research to implement the intervention in two general practices in the North of England was undertaken. The intervention combined two different participatory practices: *partnership working* involving externally facilitated meetings with PPG members and staff; and *consultation* with the wider patient population using a bespoke discrete choice experiment (DCE).

**Method:**

To illustrate decision making in PPGs, qualitative data are presented from participant observation notes and photographed visual data generated through participatory methods. The DCE results are summarised to illustrate how wider population priorities contributed to overall decision making. Observational data were thematically analysed using normalisation process theory with support from a multi-stakeholder co-research group.

**Results:**

In both general practices, patients influenced decision making during PPG meetings and through the DCE, resulting in bespoke patient-centred action plans for service improvement. Power asymmetries were addressed through participatory methods, clarification of PPG roles in decision making, and addressing representational legitimacy through wider survey consultation.

**Conclusion:**

Combining participatory practices and facilitated participatory methods enabled patients to influence decision making about general practice service improvement. The policy of mandatory PPGs needs updating to recognise the need to resource participation in a meaningful way.

## Introduction

International primary care policies have promoted patient participation in decision making about health care for over half a century.^1^^–^^4^ These policies recognise patients’ rights to involvement in decision making about both their individual clinical care and service design.^5^^–^^7^ Although there is significant literature on individual clinical shared decision making,^8^^,^^9^ the terminology, meaning, and purpose of participation in service design remains contested and variably interpreted.^3^^,^^10^^–^^12^ Despite this, patients’ right to be involved is now enshrined in the UK NHS Constitution,^6^ and since 2015 enacted in English general practices through contractual requirements to engage with patients in patient participation groups (PPGs).^13^

PPGs have existed in England since the 1970s; however, there are concerns about their effectiveness and value.^14^ One small randomised controlled trial (RCT) in 2006 found no effect of having a PPG on patient experience.^15^ But the follow-up period was short, and qualitative evaluation identified patient-centred improvements in practices with PPGs compared with those without. Other research suggests confusion about the purpose of PPGs, the meaning of legitimate representation, and barriers related to organisational culture, professional power, and social norms around the doctor–patient relationship.^16^ Internationally, short-term interventions have attempted to involve patients in decision making about the organisation of general practice.^17^^–^^19^ All highlighted similar issues around legitimate representation and facilitating power between patients and staff. A Canadian RCT and process evaluation conducted within a real-world priority-setting exercise combined different participatory practices to legitimise public knowledge and representation, and external facilitation to enable the public to influence power.^20^^,^^21^ Public participation increased prioritisation of patient-centred quality indicators. However, the context was a regional health authority and therefore less relevant to the general practice service setting.

**Table:** How this fits in

In England, patient participation groups are the mandatory mechanism for involving patients in service improvement decision making, but there is little research on how to do this. An intervention was implemented that combined two different participatory practices: 1) *partnership working*, using facilitated meetings and participatory methods; and 2) *consultation* with an adaptable prioritisation survey. Patients influenced decision making, championing patient-centred service improvement priorities that were actionable in their local general practice. For the policy of mandatory patient participation to succeed, there needs to be more attention to the process, external facilitation, adequate resourcing, and participatory methods focused on equity of voice.

To date, to the authors’ knowledge, no systematic approach has analysed and addressed the impact of representational legitimacy and power sharing in English PPGs. The authors therefore co-designed a theoretically informed intervention to support patients to influence decision making about priorities for service improvement in general practice.^16^ The focus was explicitly on service improvement decisions aligned with the contractual purpose of PPGs.^13^ This paper reports how the intervention was enacted – specifically, who made what decisions, how PPG members and staff understood their roles as decision makers, the role of participatory methods in decision making, and how final action plans were generated.

## Method

### Study design

This participatory action research study took place in two general practices in the North of England that implemented the intervention. Participatory research takes an explicit collaborative approach where the ‘researcher’ and ‘subject’ have equal ownership of the knowledge created.^22^^,^^23^ A co-research group, comprising seven members of the public with different experiences of PPGs and two GPs (all authors of this paper), was involved in all aspects of the research.

### Intervention

The intervention, detailed in Supplementary Table S1, comprised two participatory practices as defined by Arnstein.^24^ Arnstein’s ladder of citizen participation was chosen explicitly for its focus on the variable (re)distribution of power and the recognition that, without this, participation can be frustrating for the powerless and maintain the *status quo*. The ladder categorises eight different participatory practices according to the power that citizens have to influence change.^24^ The intervention focused on and combines only two of these practices. This is because there is increasing recognition that combining participatory practices can address issues raised by the complexity of public service governance and the heterogeneity of citizen preferences for participation.^25^^,^^26^ The two participatory practices were:

- *partnership working* where power is redistributed so that citizens (PPG members) share decision-making power and responsibility with those in established authority positions (general practice staff); and
- *consultation* where citizens (patients) are consulted about their opinions on pre-defined categories chosen by those with power and who will decide whether and how to act on the opinions expressed (both PPG members and staff).

In the intervention, *partnership working* was enacted through facilitated meetings to support PPG members and staff to share decision making. Initially two single stakeholder and one mixed stakeholder meetings focused on choosing five features of their service they would be willing to change. Meetings utilised external facilitation and a range of participatory methods: card-sort, direct ranking, and flexible brainstorm.^27^^–^^29^ These were adapted from Participatory Learning and Action tools that promote equity of voice and knowledge sharing between stakeholders to address power asymmetries.^29^^–^^33^

*Consultation* was enacted through a bespoke discrete choice experiment (DCE) survey to consult each practice’s patient population preferences for service improvement from those chosen by the PPG members and staff. DCEs force responders to make choices between hypothetical service alternatives as opposed to methods that involve responders rating individual services separately.^34^^,^^35^ The DCE aimed to broaden patient participation in decision making and strengthen representational legitimacy.

*Partnership working* then focused on agreeing a bespoke action plan for service improvement in a final mixed stakeholder meeting.

### Site selection, sampling, and recruitment

General practices were identified by combined convenience and purposive sampling based on: openness to change; enthusiasm for the project; having an existing functioning PPG; and location in an area of lower socioeconomic status. Practices were excluded if any co-research group member, including the lead clinician–researcher (the first author), was either a member of the PPG or practice staff.

In each practice the co-research group liaised with a gatekeeper who facilitated access and distributed participant information sheets, consent forms, and intervention details to PPG members and staff. Both sites had existing PPGs with established members recruited via a variety of approaches by the general practices, as is usual practice. All participants in intervention meetings gave signed consent. Practices were paid £750 for participation in the research. In keeping with norms in English general practice, PPG members were not paid for their time, but were made aware of the practice payment.

### Data collection and analysis

Two co-research group members (the first author and a member of the co-research group) co-facilitated every intervention meeting and observed one to three follow-up meetings, for up to a year. These external participant observer roles allowed for interaction and to facilitate constructive dialogue.^36^ Data included participant observation notes, photographed visible data regarding the decisions made using the participatory methods (results of the card-sort, voting, and flexible brainstorm), and the outcome of the DCE, summarised here to illustrate how individual patients influenced decision making outside the PPG.

Observational notes focused on how decisions were made and by who within intervention meetings, and the relationships and interactions between patients and staff with specific attention to representational legitimacy and power sharing. A formal framework was not used for observational notes as the co-research group found this too restrictive. Observational notes were made during observed meetings and then typed up and expanded later.

Thematic data analysis of observational data was iterative and began after each meeting with a reflexive debrief between the first author and the co-research group member who co-facilitated the meeting.^37^ Observational notes were then shared with the whole co-research group and discussed in detail during multiple co-analysis workshops. These discussions happened during ongoing data collection with a constant comparison approach.^36^^–^^38^

Analysis was deductive using normalisation process theory (NPT) to explore the work required to implement the intervention, with particular attention to disconfirming data relevant to representational legitimacy and power.^39^ NPT is a sociological theory that evaluates the work of individuals and groups to introduce a new way of working (the intervention) into a healthcare setting (PPGs in general practice). Following early co-analysis workshops, the first author produced an initial coding framework incorporating all themes that was then discussed and refined in further regular co-analysis workshops after completing data collection. The first author coded all observational notes using NVivo with regular checking with the co-research group.

## Results

Two of six general practices approached agreed to participate. Four practices declined because of concerns about their overall workload and/or PPG commitment. Both recruited practices were located in areas of lower socioeconomic status (the third and second most deprived deciles). Most PPG members were over 50 years old. In Practice 1, PPG members were split almost evenly between White and Black ethnicity. In Practice 2, all participants were White. Supplementary Table S2 summarises practice and PPG characteristics.

Twenty-nine patients and 36 members of staff took part in at least one intervention meeting (Supplementary Table S3). In both practices a core group attended all meetings, whereas the rest only attended one meeting. At least two members of staff attended every meeting, usually the practice manager and one GP. Staff participants included GPs (partners, salaried GPs, and trainees), nurses, administrators, managers, and receptionists.

The results are reported in two sections: first, who made what decisions drawing on the results of the participatory methods and prioritisation survey; and second, how decisions were made.

### Who made what decisions?

The first three facilitated meetings supported PPG members and staff to share decisions about which five features, from a list of 24 rigorously designed features (Supplementary Table S4),^16^ to include in their prioritisation survey.

In the first meeting, PPG members and staff participated in a card-sort to choose features they were interested in and believed were feasible to change. Levels of agreement varied between stakeholder groups and across practices (see Supplementary Table S5; discussed further below). In each practice, either PPG members or staff judged 20 (Practice 1) and 16 (Practice 2) features as feasible to change.

In the second meeting, PPG members and staff met together to vote for five features to include in the final survey from those judged feasible to change. They voted individually, then discussed their votes, then voted individually again with the combined top-scoring features included in the survey. In both practices, everyone changed at least one vote in the second round of voting, resulting in differences in the top five features between voting rounds (detailed in Supplementary Tables S6 and S7). Selected final features did not overlap across the two practices.

The top five features in each practice were adapted into a bespoke DCE prioritisation survey; 333 and 343 surveys were completed respectively in Practices 1 and 2. In both practices the online survey produced the highest number of responses, followed by the paper survey, and then ballot box survey. Response rates are only available for the online and paper surveys as the ballot box survey was left out in the waiting room with no mechanism to monitor how many people saw it and did not complete the survey (see Supplementary Table S8 for responder characteristics).

Compared with limited nationally published practice demographic data, responders were more likely to be female, White, and university educated (socioeconomic status used as a proxy comparison).^40^ In both practices, the paper survey produced the most diverse sample.

Practice 1 patients most valued the feature ‘How well the doctors and nurses listen and pay attention to you’. Practice 2 patients most valued ‘How long your appointment lasts’. Table 1 shows the order of preference for the different features (detailed results available elsewhere).^16^

**Table 1.** Order of preference of five features following the prioritisation surveys

**Ranking**	**Practice 1**	**Practice 2**
**First**	9: How well the doctors and nurses listen and pay attention to you^a^	1: How long your appointment lasts^a^
**Second**	10: How involved you are in making choices about your care^a^	15: How well your doctor or nurse knows your medical history^a^
**Third**	23: How the patient support staff treat you^a^	17: How often you get your choice of doctor and nurse
**Fourth**	19: How many services are offered by the practice^a^	14: How you are supported to manage your own health
**Fifth**	30: How the staff respond to feedback and complaints^a^	12: How often community groups and lifestyle activities are suggested

a *Results that were statistically significant. See reference 16 for details.*

Around half of survey responders left free-text comments: 159 of 333 (47.7%) in Practice 1, and 179 of 343 (52.2%) in Practice 2. These comments related to the features in each survey and identified additional priorities for change (Supplementary Table S9).

Following the survey, PPG members and staff met to participate in a flexible brainstorm exercise to agree on relevant practical actions. This process generated specific action plans for each practice. Actions were based on a number of sources: the quantitative DCE survey results, qualitative free-text responses, and meeting deliberations (Box 1).

**Box 1.** Action plans for Practices 1 and 2

**Practice**	**Actions**		
**1**	**Improving communication with the patient population^a^** Including: raise awareness of what the practice offers^a^ with support from PPG^b^	**Ethnicity and improving patient experience^b^** Including: investigate and act on differences by working with local community groups^b^	**Maximising patients feeling listened to^c^** Including: non-violent communication skills training for staff;^b^ improve continuity of care;^d^ help patients prepare for appointments^b^
**2**	**Improving the appointment system and experience of booking appointments^c,d^** Including: improve privacy in reception area;^d^ raise awareness of services with patients and staff;^d^ change appointment system including appointment length^c,d^	**Supporting patients to manage their own health^a^** Including: raise awareness of local community resources via noticeboards and clinicians;^a^ set up peer support groups;^b^ group consultations^b^	**Making the patient group more accessible^b^** Including: advertise the group better; change the name; explore different meeting times)

a *Feature included in DCE but not highly ranked.*

b *Idea originated from meeting participant.*

c *Feature highly ranked in DCE.*

d *Idea originated as qualitative free-text survey response. DCE = discrete choice experiment. PPG = patient participation group.*

Practice 1 started to implement its whole action plan. Staff agreed that they might have acted to improve communication without the intervention, but all the other actions were because of the intervention.

Practice 2 did not implement its action plan because of the COVID-19 pandemic.

### How were decisions made?

#### How PPG members and staff understood their roles as decision makers

Holding the card-sort exercise as two separate stakeholder meetings allowed both groups to explore their role in decision making. All features generated discussion by both PPG members and staff, and there were similarities and differences in the decisions they made about which features to include in the survey.

Staff were more confident of their decision-making role and rejected more features than patients, usually because they felt changing them was beyond their control:

*‘*[Feature 5: When you can have an appointment] [Participant (P)12, GP partner] *immediately said “I don’t want that in there.” She clarified saying there is no way that they are going to increase the hours that they provide outside nine–five, and* […] *“we are not going to change”.’*

(Practice 1, Meeting 1b, staff-only card-sort exercise)

*‘*[Feature 19: How many services are offered by the practice] [P7, the practice manager] *said this was a contractual issue (and therefore couldn’t be changed) and they are providing all the services they are contractually required to provide.* […] [P7, the practice manager] *definitively, said “no options to increase — red”.’*

(Practice 2, Meeting 1b, staff-only card-sort exercise)

PPG members were unaware of some features and did not always know what current practice was, for example, Feature 20: ‘How much patients are charged for requests for letters of support’, Feature 21: ‘How interpretation services are provided’, and Feature 6: ‘How easy is it to get a home visit’. This lack of experiential knowledge resulted in uncertainty about their role in decision making and perceived illegitimacy regarding the power to represent the views of other patients:

‘[Feature 6: How easy is it to get a home visit] [P3, PPG member] [said] *“Ooohhh, interesting.” There was then a pause whilst they all looked at each other. Then* [P2, PPG member] *and* [P1, PPG member] *said that they had no idea how to get a home visit or how easy it was. There was another pause, then* [P2, PPG member] *remembered requesting a home visit a couple of years ago.* […] *She finished* [the story of her experience] *by suggesting that it would be interesting to put* [Feature 6] *to the patient body. This felt like a suggestion because the group didn’t have enough experience of it as an issue.’* (Practice 2, Meeting 1a, PPG member-only card-sort exercise)

*‘The consensus of the group appeared to be that* [Feature 10: How involved you are in making choices about your care] *was important but managed quite well at the practice. However, someone* […] *said “Would you want this group to speak on your behalf?” and then everyone agreed it would be better to find out whether the wider practice population thought* [Feature 10] *was an issue. This set the tone for decision making going forward.’* (Practice 1, Meeting 1b, staff-only card sort exercise)

#### How participatory methods legitimised decision-making roles

Participatory voting in mixed stakeholder groups, with everyone having the same weight of vote, demonstrated that the voice, and hence power, of all those present was valued equally. The discussion allowed sharing of staff organisational knowledge and patient experience knowledge, clarifying and legitimising all stakeholders’ roles and value in a decision-making process. Staff in both practices had the opportunity to explain why changing certain features was not possible; this inferred task legitimacy on the voting about items to include in the survey:

*‘*[P14, the practice manager] *said very clearly it would not be possible to change when people can get appointments in the near future. Therefore there was no point in asking patients about this, as it would just raise expectations.* [P18, PPG member] *who had been very passionate about this feature in the card-sort, said “OK I see your point and I agree there is no point having it in.”’*

(Practice 1, Meeting 2, voting in a mixed group of PPG members and staff)

Perceived role legitimacy activated PPG members who championed certain features. In Practice 1 this resulted in these features getting more votes in the second round:

*‘*[P20, PPG member] *said that the key* [feature] *for her was about how receptionists treat you* [Feature 23]*. Because this is the front end of the practice and the first bit people encounter.* [The first author asked] *“Is this something you can really change?”* [P14, the practice manager] *came back at* [the first author] *and said that yes it was the perfect timing for this, because the receptionists are taking on more signposting roles and they want to know what patients think, and make sure receptionists are adequately trained to know how to do this in a supportive way.’*

(Practice 1, Meeting 2, voting in a mixed group of PPG members and staff)

In both practices, the facilitated sharing of dialogue about different knowledge fostered mutual understanding of differing perspectives. Rather than conflict, it resulted in everyone changing their votes between the first and second rounds. This was demonstrated in Practice 2 after the first round of voting when P14, a PPG member, championed improving privacy around the open waiting room reception desk. In return P9, a receptionist, showed empathy:

*‘*[P9: receptionist] *said that she was sorry* [P14, PPG member] *felt the reception area wasn’t private, and that “If you ever need privacy you can tell the receptionist, and there is a quiet area around the corner where you can speak privately.” This spontaneous response didn’t feel defensive.’*

(Practice 2, Meeting 2, voting in a mixed group of PPG members and staff)

After this interaction, in the second round of voting P14 did not give any votes to Feature 24 ‘Privacy at reception’ because she had been told there was a solution.

#### How the final action plans were generated

In both practices the action plans were generated from several sources (Box 1). Some participants (both PPG members and staff) lacked confidence in interpreting the quantitative survey results given their complexity. However, all were still willing to participate in action planning and features rated highly in the survey were seen as legitimate priorities for service improvement by both PPG members and staff:

‘[P20, PPG member] *said she would like to work on people feeling listened to enough. A lot of other people* [also identified this priority]*, including* [P12, the GP partner]*.*’

(Practice 1, Meeting 3, flexible brainstorm in a mixed group of PPG members and staff)

Features only mentioned in the survey free-text responses were also seen as legitimate priorities because they highlighted previously unknown or unacknowledged concerns. For example, in Practice 2 there were many emotive free-text comments about the lack of privacy at the waiting room reception desk. This privacy issue was discussed in the voting meeting, but staff suggested the problem had been addressed (see above). Following the free-text comments, they realised their solution was not working and it re-emerged as a priority:

*‘*[P7, the practice manager] *had one* [suggestion] *about privacy at reception. He said he hadn’t realised what it was like, and since reading the free-text comments had been much more aware of the issues in the reception area.’*

(Practice 2, Meeting 3, flexible brainstorm in a mixed group of PPG members and staff)

Features included in the survey but low scoring (Feature 19: ‘How many services are offered by the practice’ in Practice 1, and Feature 12: ‘How often community groups and lifestyle activities are suggested’ in Practice 2), and features not mentioned in the survey at all, were also included in the action plans through being championed by people present in the meeting (both PPG members and staff), especially if they had experiential knowledge of a feature:

*‘*[P15, PPG member] *started by saying “I live on my own and I’m depressed”, she said that knowing about local community groups would have really helped her and therefore she would like to see self-help groups publicised more.* […] [P3, PPG member] *and* [P7, the practice manager] *also had similar suggestions about the need to raise awareness of local community groups, self-management, and social prescribing.’*

(Practice 2, Meeting 3, flexible brainstorm in a mixed group of PPG members and staff)

## Discussion

### Summary

Combining participatory practices — partnership working and consultation^24^ — and using facilitated participatory methods^27^^–^^29^ supported PPG members and staff to understand that they both had legitimate roles as decision makers, helped to address power asymmetries, and increased representational legitimacy. PPG members shared their experiential knowledge of services and staff shared their practical knowledge of service improvement. The exchange of credible knowledge during participatory voting resulted in everyone changing their choices for features to include in the survey, sometimes in favour of PPG members and sometimes in favour of staff. The wider patient population were able to share their opinions via consultation in the survey. Survey responders were generally atypical of the practice profile; however, PPG involvement in survey distribution enhanced sample diversity, increasing representational legitimacy. Although action plans were not solely based on survey data, PPG members were present in discussions about the interpretation and use of these data in bespoke patient-centred action plans. Thus, these plans were still heavily patient influenced.

### Strengths and limitations

This is the first evaluation, to the authors’ knowledge, of a systematic approach to enable patients to influence organisational decision making in English general practice. In both practices PPG members and staff engaged in the facilitated meetings and the patients (as PPG members and by completing the survey) contributed to decision making. However, this resource-intensive process happened in only two self-selecting practices and required external facilitation; further testing is needed to assess costs and applicability.

The use of a DCE as a locally adaptable consultation tool to stimulate individual general practice service improvement is novel. There was no overlap in the five features chosen for the survey in each practice, highlighting the need for a locally adaptable survey. The survey appears to deliver sufficiently precise results within the wider intervention to stimulate change by providing representational legitimacy, despite survey responder profiles being less diverse than the practice populations.

### Comparison with existing literature

Evaluations of attempts to increase patient influence in decision making highlight the importance of representational legitimacy. In Canadian family practices, patients and staff working in small action research groups suggested collecting survey data to overcome representational deficit.^19^ In one Canadian regional health authority, patients who incorporated survey data into their discussions with staff gained representational legitimacy.^20^ Similarly, the current study found PPG members initially struggled making decisions on others’ behalf and only became more confident with their role when drawing on representationally legitimate survey data to construct improvement action plans. However, overall, action plans were only partly based on the survey results. In hospital settings, staff only acted on patient feedback if they believed they had the agency and resources to effect change and the organisation was able to change.^41^ The authors of the current study also observed staff limiting what could be included in the survey, and thus changed, based on their beliefs about their agency and resources to effect change. Therefore, staff input into survey development ensured actionable results, albeit sometimes at the expense of patient priorities such as privacy at the reception desk (initially) in Practice 2. Action plans were also partly based on free-text responses and individuals’ own ideas. Such ‘soft intelligence’ can help the early recognition and prevention of poor care.^42^ In Practice 2, free-text qualitative data resulted in ‘privacy’ re-emerging as a priority. Similar to other research,^20^^,^^21^ this demonstrates the interaction, and interdependent relationship, between stakeholder participation in credible deliberation within meetings (partnership working) and the representative quantitative and qualitative survey data (consultation) to achieve patient influence and to generate feasible action plans.

Literature on individual clinical decisions has identified important components of shared decision making.^8^^,^^9^^,^^43^^,^^44^ These include creating choice awareness, information sharing, and elicitation of values and preferences, all through a deliberative approach. This intervention included these components: presenting lesser-known features of general practice created choice awareness; joint meetings enabled sharing of patient experience and staff organisational knowledge; and voting discussions elicited different and complementary values and preferences. This suggests these components are also important for organisational shared decision making.

In individual decision making, satisfaction increases if people experience a supportive deliberative decision-making process, even for cognitively challenging decisions that elicit negative emotions.^43^^,^^44^ PPG members initially found decision making uncomfortable, but gained confidence over the course of the intervention. This appears to be because of external facilitation and participatory methods that promoted equitable contributions and addressed power, creating a supportive deliberative process. Therefore, as with individual decision making, how decisions are made can be as important as what decisions are made. These findings resonate with other research highlighting the importance of attention to the process of participation,^29^ combining different participatory practices,^20^^,^^21^^,^^25^^,^^26^ and comparing individual and collective forms of decision making and participation.^2^

### Implications for research and practice

Different participatory practices can be combined to support patients to influence organisational decision making in general practice. The intervention needs testing in more practices, with a longer follow-up to evaluate the normalisation of PPGs in decision making, and the effect on patient-centred services and care. Further research could test different models of facilitation of partnership working, and whether simpler consultation methods, such as best–worst scaling or participatory ranking methods, might be more sustainable.^27^^,^^34^^,^^45^

The current English general practice contractual requirement to have a PPG is an important lever for patient participation, but this policy neither encourages nor supports the necessary participatory practices for its meaningful enactment. Policy needs to recognise participation requires planning, facilitation, and adequate resources. Recent policies have suggested public participation at the level of primary care networks and that this will help to address health inequalities.^46^ The research in the current study suggests that this will not happen by default and that the process of participation is as important as the outcome. Combining well-resourced and legitimate participatory practices fosters transparency and builds trust between both patients and staff. Given that trust in the profession is falling and staff feel undervalued^47^^,^^48^ there is a strong case for investment in meaningful patient participation now more than ever.

## Data

The data sets are not publicly available.
